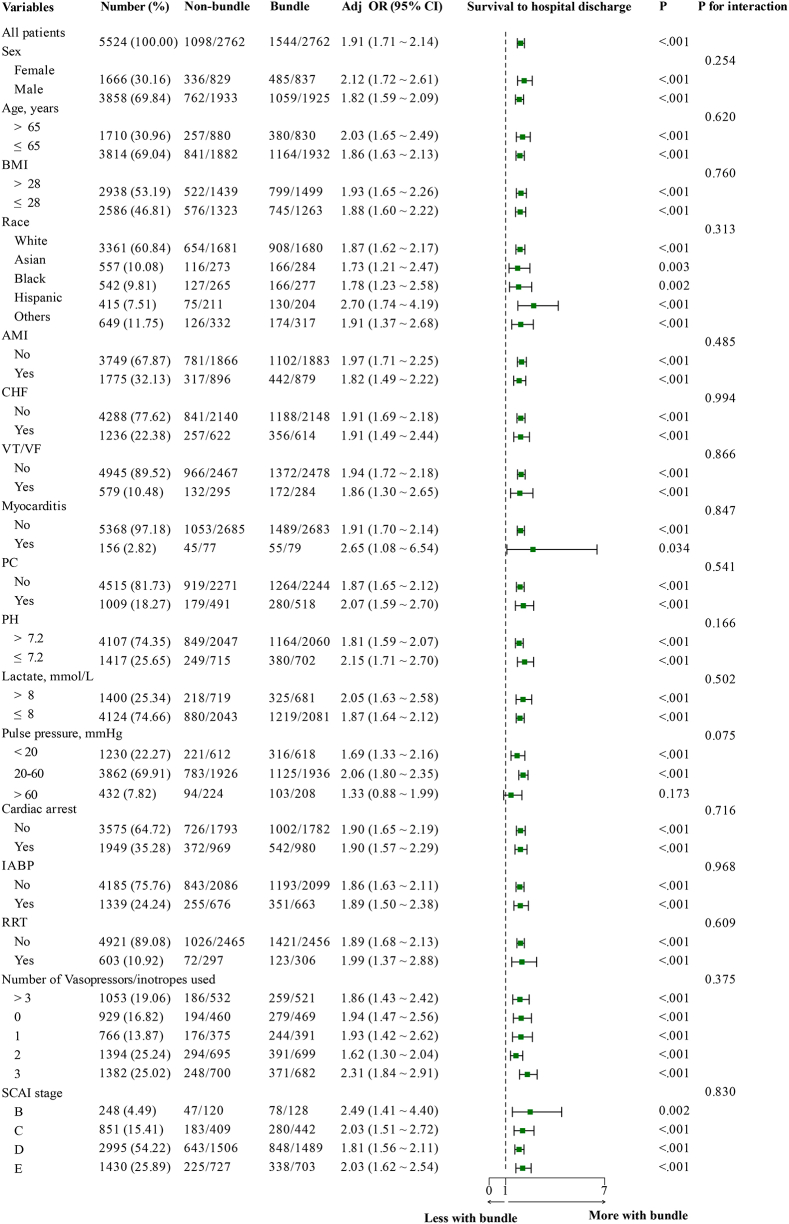# A simple VA-ECMO bundle in adult patients with cardiogenic shock: an analysis of ELSO registry

**DOI:** 10.1016/j.eclinm.2025.103423

**Published:** 2025-08-07

**Authors:** Liangshan Wang, Kexin Wang, Yan Wang, Feng Yang, Chenglong Li, Xing Hao, Zhongtao Du, Peter T. Rycus, Joseph E. Tonna, Eddy Fan, Hong Wang, Xiaotong Hou

**Affiliations:** a Center for Cardiac Intensive Care, Beijing Anzhen Hospital, Capital Medical University, Beijing, People's Republic of China; b Extracorporeal Life Support Organization (ELSO), University of Michigan, Ann Arbor, MI, USA; c Division of Emergency Medicine, Division of Cardiothoracic Surgery, Department of Surgery, University of Utah Health, Salt Lake City, UT, USA; d Interdepartmental Division of Critical Care Medicine, University of Toronto, Toronto, Ontario, Canada

**Keywords:** Veno-arterial extracorporeal membrane oxygenation, Cardiogenic shock, Simple bundle, Extracorporeal Life Support Organization registry

## Abstract

**Background:**

The optimal management strategy for patients undergoing veno-arterial extracorporeal membrane oxygenation (VA-ECMO) for cardiogenic shock (CS) remains uncertain. To evaluate the impact of “bundled” physiologic targets on outcomes in VA-ECMO patients.

**Methods:**

This retrospective cohort study analyzed data from the Extracorporeal Life Support Organization (ELSO) registry, including adult patients receiving VA-ECMO for CS between 2013 and 2022. Patients were grouped by whether they received the full set of bundle components within the first 24 h. The bundle included mean arterial pressure > 65 mmHg, PaO_2_ 60–150 mmHg, relative change in PaCO_2_ [RelΔCO_2_] > −50% (RelΔCO_2_ = [(PaCO_2-24hours_−PaCO_2-baseline_)/PaCO_2-baseline_] ∗ 100%), and peak inspiratory pressure < 30 mmHg. The primary outcome was survival to hospital discharge, while secondary outcomes were complications.

**Findings:**

Of 7950 patients (mean age 56.5 ± 14.3 years), 2762 (34.7%) received the complete bundle. The bundle group had significantly higher rates of survival to hospital discharge (55.9% vs. 39.4%, *p* < 0.001) with adjusted odds ratio [aOR] of 1.849 (95% CI [1.675, 2.042]; *p* < 0.001), and the likelihood of brain death (aOR = 0.521, 95% CI: 0.289–0.885; *p* = 0.021), ischemic stroke (aOR = 0.710, 95% CI: 0.559–0.896; *p* = 0.004), hemorrhagic complications (aOR = 0.773, 95% CI: 0.681–0.876; *p* < 0.001) and cardiovascular complications (aOR = 0.863, 95% CI: 0.770–0.966; *p* = 0.011) were reduced.

**Interpretation:**

Achieving the proposed physiologic bundle is associated with improved survival and a reduced risk of complications in VA-ECMO patients with CS. These findings provide evidence supporting the use of standardized care bundles in the management of VA-ECMO patients with CS.

**Funding:**

This work was supported by the Beijing Hospitals Authority Clinical Medicine Development of Special Funding Support (No. ZYLX202111, to X Hou), Beijing Hospitals Authority “Ascent Plan” (No. FDL20190601, to X Hou), Young Elite Scientists Sponsorship Program by CAST (No. 2022QNRC001, to L Wang), 10.13039/501100001809National Natural Science Foundation of China (No. 82200433, to L Wang), and Beijing Hospitals Authority Youth Programme (No. QML20230602, to L Wang), 10.13039/501100001809National Natural Science Foundation of China (No. 82100408, to X Hao), 10.13039/501100005090Beijing Nova Program (No. 2022064, to C Li), 10.13039/501100005089Beijing Natural Science Foundation (No. 7244327, to C Li), and the 10.13039/501100012166National Key Research and Development Program of China (No. 2021YFC2701700 and 2021YFC2701703, to Z Du).

**Research in context**

**Evidence before this study**

We searched the PubMed, MEDLINE, Embase and Cochrane databases for the terms “extracorporeal membrane pulmonary oxygenation”, “cardiogenic shock”, “blood pressure”, “oxygenation”, “carbon dioxide” and “mechanical ventilation” from inception to December 31, 2024. Previous analyses of the Extracorporeal Life Support Organization (ELSO) registry have shown that hyperoxia, relative change in PaCO_2_, and high peak inspiratory pressures are individually associated with a poor prognosis in patients with ECMO. However, no study has evaluated these parameters as a combined bundle approach in VA-ECMO patients with cardiogenic shock.

**Added value of this study**

This first large-scale study evaluated a comprehensive physiological bundle (mean arterial pressure >65 mmHg, PaO_2_ 60–150 mmHg, relative change in PaCO_2_ > −50%, and peak inspiratory pressure < 30 mmHg) within 24 h of VA-ECMO initiation. Among 7950 ELSO registry patients, complete bundle achievement (34.7% of patients) was associated with significantly higher survival (55.9% vs. 39.4%; adjusted OR 1.849, 95% CI: 1.675–2.042) and lower rates of brain death, stroke, and hemorrhagic complications. These findings suggest potential benefits of the simple bundle in VA-ECMO management.

**Implications of all the available evidence**

The results suggest that implementing a standardized physiological bundle during early VA-ECMO support may help improve outcomes in cardiogenic shock patients. Future research should consider prospective validation, evaluation of individual component contributions, and strategies for improving clinical adherence. With further research this bundle could potentially serve as a practical intervention to standardize care in VA-ECMO patients with cardiogenic shock.

## Introduction

Veno-arterial extracorporeal membrane oxygenation (VA-ECMO) has expanded rapidly as a salvage strategy to provide temporary circulatory and respiratory support allowing cardiac recovery or bridge to additional therapeutic alternatives in patients with cardiogenic shock (CS).^1^, ^2^, ^3^ However, important aspects of extracorporeal membrane oxygenation (ECMO) management, including blood pressure target, oxygenation, carbon dioxide, and mechanical ventilation targets remain unclear. In non-ECMO shock, an evidence-based target of 65 mmHg has been recommended to sustain end-organ function.^4^ Previous studies using Extracorporeal Life Support Organization (ELSO) Registry data have demonstrated that hyperoxia,^5^ relative drop in partial pressure of carbon dioxide (PaCO_2_) >50%,^6^^,^^7^ peak inspiratory pressure (PIP) > 30 mmHg^8^ were associated with increased in-hospital mortality or neurological risks in patients undergoing ECMO. In this context, we define a simple ECMO bundle during the first 24 h following VA-ECMO initiation based on available data from ELSO registry, including achieving the following targets: mean arterial pressure target (MAP) > 65 mmHg, oxygenation (partial pressure of arterial oxygen [PaO_2_] 60–150 mmHg), carbon dioxide (relative change in PaCO_2_ [RelΔCO_2_] > −50%, RelΔCO_2_ = [(PaCO_2-24hours_−PaCO_2-baseline_)/PaCO_2-baseline_] ∗ 100%), and lung protective ventilation (PIP < 30 mmHg). We hypothesized that achieving a simple bundle of early physiological targets within 24 h of VA-ECMO initiation would be associated with improved in-hospital survival and reduced complications in adult patients with CS.

## Methods

### Population

The ELSO registry is a voluntary international registry of patients treated with ECMO that by 2022 included 577 centers.^9^ Patient characteristics, pre-ECMO interventions, ECMO circuit details, adverse events, and outcomes are recorded using a standardized data collection form. We analyzed ECMO patient-run data from the ELSO Registry to identify adult patient-runs who received VA-ECMO for CS between 2013 and 2022. CS was defined using International Classification of Diseases (ICD)-9/10 codes (785.51 and R57.1, respectively). Exclusion criteria were: (1) more than 1 run of VA-ECMO, (2) received an ECMO mode other than VA, (3) discharged on VA-ECMO, and (4) missing data for any of the following key bundle related variables: PaCO_2_, PaO_2_, PIP, and MAP at 24 h on VA-ECMO and pre-ECMO PaCO_2_.

### Ethics

This study was approved by the Beijing Anzhen hospital Institutional Ethics Committee (2025146x). Informed consent for demographic, physiological and hospital-outcome data analyses was waived because this observational study did not modify existing diagnostic or therapeutic strategies.

### Determination of bundle criteria

The physiological bundle evaluated in this study was developed through a three-step approach that combined literature evidence, expert consensus, and statistical validation. We first conducted a targeted literature review to identify physiological parameters previously associated with improved outcomes in VA-ECMO or critically ill patients, focusing on MAP, PaO_2_, PaCO_2_, and PIP. Based on current guidelines and pathophysiological rationale, the study defined as the bundle included the following variables and corresponding value ranges, measured at 24 h of ECMO: MAP > 65 mmHg, PaO_2_ 60–150 mmHg, RelΔCO_2_ > −50%, and PIP < 30 mmHg. These components were then validated using a Bayesian logistic regression model applied to the ELSO registry dataset, which confirmed their individual associations with survival. Further details on the machine learning Bayesian model are described in the Supplementary Methods. Patients were subsequently divided into a bundle group, who achieve all four criteria within 24 h of ECMO initiation, and a non-bundle group, who did not achieve one or more of the four components. Of note, RelΔCO_2_ was a variable of interests the change in PaCO_2_ from 6 h prior to ECMO until 24 h of ECMO, calculated as follows: [(PaCO_2-24hours_−PaCO_2-baseline_)/PaCO_2-baseline_] ∗ 100%.

### Outcome and definitions

The primary outcome was survival to hospital discharge. Secondary outcomes included the following complications categories. Individual complications for each category were listed in the Supplementary Methods.

### Statistics

All statistical analyses were performed using SPSS (version 19.0; SPSS Inc., Chicago, IL, USA) and R 4.3.1 (http://www.R-project.org).

#### Descriptive statistics

Continuous variables are reported as means and standard deviations; categorical variables, are reported as frequencies and proportions. Comparisons of continuous variables were performed using the Student t test or the Mann–Whitney U test, and categorical data were analyzed using the Fisher exact test or the Pearson chi-square test. *p*-values less than 0.05 were considered statistically significant.

#### Missing data handling

Patients with missing data on key bundle components (pre-ECMO PaCO_2_, PaCO_2_ at 24 h, PaO_2_ at 24 h, PIP at 24 h, or MAP at 24 h) were excluded from the analysis. For other variables, missing data were imputed using the multiple imputation by chained equations (MICE) method, assuming missing at random (MAR). In this context, MAR implies that the probability of a variable being missing depends only on observed data, but not on the missing value itself. A total of five estimation datasets were generated and the results were summarized to obtain valid estimates of the model parameters. To assess the robustness of results, we compared the complete case analysis with the multiply imputed analysis (Supplementary Table S5).

#### Multivariable logistic regression

Multivariable logistic regression was used to evaluate the association between Survival to hospital discharge and complication rates of bundle. Adjusted odds ratios were determined after adjusting by age, sex, race, weight, CS-related diagnosis (including acute myocardial infarction [AMI], congestive heart failure [CHF], ventricular tachycardia/ventricular fibrillation [VT/VF], myocarditis, and post-cardiotomy [PC]), pre-ECMO cardiac arrest (CA), pre-ECMO renal replacement therapy (RRT), intra-aortic balloon pump (IABP), pre-ECMO pH, pre-ECMO lactate, pulse pressure at 24 h, and Society for Cardiovascular Angiography & Interventions (SCAI) staging, which has been shown to correlate with survival in patients treated with ECMO.^10^ The covariates included in the multivariable model were selected a priori based on their known or plausible associations with both the exposure (bundle achievement) and the outcome (in-hospital mortality), as supported by previous literature on VA-ECMO patient management. These variables encompassed demographics, clinical severity, pre-ECMO interventions, and comorbidities.

#### Sensitivity analyses

Adjusted logistic regression models were used for sensitivity analyses to estimate the relationship between bundle and survival to hospital discharge in different subgroups. Selected continuous variables were dichotomized based on established clinical thresholds or previously published cut-off values. Subgroups were defined according to sex, age (>65 and ≤65 years), obesity status (BMI > 28 and BMI ≤ 28), race (White, Asian, Black, Hispanic, and others), and diagnoses related to CS (AMI, CHF, VT/VF, myocarditis, and PC), pre-ECMO lactate (>8 mmol/L and ≤8 mmol/L), pre-ECMO pH (>7.2 and ≤7.2), pre-ECMO pulse pressure (<20 mmHg, 20–60 mmHg, and >60 mmHg), pre-ECMO CA, pre-ECMO RRT, IABP, vasopressor/inotropic type (0, 1, 2, 3, and >3), and SCAI staging (B, C, D, and E). Models were adjusted for age, sex, race, weight, diagnoses associated with CS, pre-ECMO CA, pre-ECMO RRT, pre-ECMO IABP, pre-ECMO pH, pre-ECMO lactate, pulse pressure at 24 h, and SCAI stage.

#### Propensity score matching

**Supplementary Figure S1:**
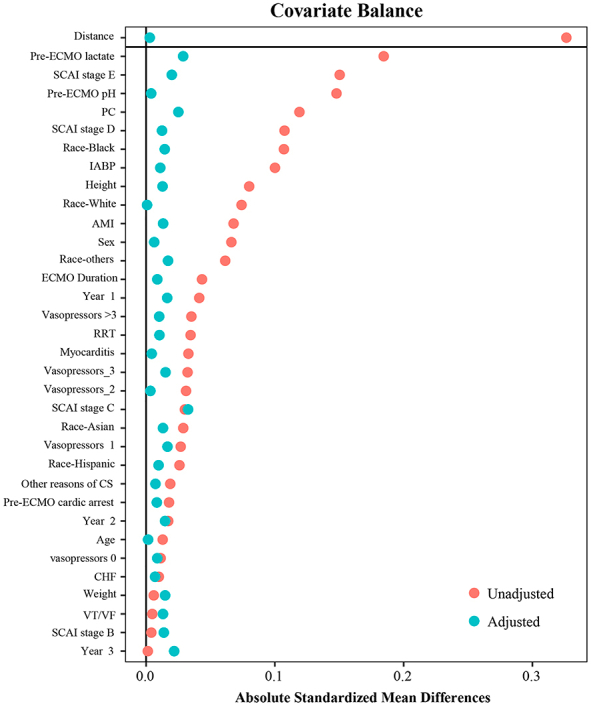


In order to balance the probability of achieving all bundle components vs. not achieving all bundle components, we preformed propensity score weighting to balance the probability of receipt of all bundle components. To address the potential for confounding in this observational dataset, the following variables were used to calculate the propensity score: age, sex, race, year, weight, height, diagnoses associated with CS, pre-ECMO CA, pre-ECMO RRT, IABP, duration of ECMO, types of vasopressors/inotropes, pre-ECMO pH, pre-ECMO lactate, and SCAI stage. Patients treated with the bundle were matched 1:1 to patients not treated with the bundle, using the nearest neighbor method without replacement. The standardized mean difference for each covariate was calculated in the propensity-matched cohort (Supplementary Figure S1 and Table 1).

**Table 1:** Demographic and clinical characteristics of the study population.

Variables	Non-bundle (N = 5188)	Bundle (N = 2762)	Overall (N = 7950)	*p*-value
Sex				0.006
Female	1730 (33.3%)	835 (30.2%)	2567 (32.3%)	
Male	3458 (66.7%)	1925 (69.7%)	5383 (67.7%)	
Age, years	56.4 (14.5)	56.6 (13.8)	56.5 (14.3)	0.816
Race				<0.001
White	2968 (57.2%)	1680 (60.8%)	4648 (58.5%)	
Asian	488 (9.4%)	284 (10.3%)	772 (9.7%)	
Black	687 (13.2%)	277 (10.0%)	964 (12.1%)	
Hispanic	348 (6.7%)	204 (7.4%)	552 (6.9%)	
Others	697 (13.4%)	317 (11.5%)	1014 (12.8%)	
Year				0.231
2013–2016	314 (6.1%)	142 (5.1%)	456 (5.7%)	
2017–2019	2075 (40.0%)	1128 (40.8%)	3203 (40.3%)	
2020–2022	2799 (54.0%)	1492 (54.0%)	4291 (54.0%)	
Weight, kg	87.4 (23.5)	87.3 (22.0)	87.4 (23.0)	0.566
Height, cm	171 (12.0)	172 (11.3)	171 (11.8)	0.001
BMI	30.1 (10.4)	29.6 (7.46)	29.9 (9.52)	0.184
AMI	1487 (28.7%)	879 (31.8%)	2366 (29.8%)	0.004
CHF	1132 (21.8%)	614 (22.2%)	1746 (22.0%)	0.695
PC	1214 (23.4%)	518 (18.8%)	1732 (21.8%)	<0.001
VT/VF	541 (10.4%)	284 (10.3%)	825 (10.4%)	0.87
Myocarditis	120 (2.3%)	79 (2.9%)	199 (2.5%)	0.158
Others	695 (13.4%)	388 (14.0%)	1083 (13.6%)	0.44
ECMO initially				
PH	7.26 (0.147)	7.28 (0.130)	7.27 (0.142)	<0.001
PaCO_2_	44.8 (23.8)	40.9 (11.0)	43.4 (20.4)	<0.001
PaO_2_	174 (138)	152 (110)	167 (129)	<0.001
Lactate, mmol/L	8.02 (5.61)	6.78 (5.03)	7.58 (5.44)	<0.001
SBP	89.6 (25.2)	90.6 (23.0)	90.2 (24.5)	0.002
DBP	55.5 (17.4)	57.2 (16.4)	56.4 (17.3)	<0.001
MAP	66.9 (17.7)	68.4 (16.9)	67.5 (17.2)	<0.001
PIP	25.8 (7.60)	24.7 (6.80)	25.4 (7.34)	<0.001
ECMO for 24 h				
PH	7.41 (0.09)	7.43 (0.07)	7.42 (0.08)	<0.001
PaCO_2_	39.9 (19.3)	38.1 (6.38)	39.3 (16.1)	0.210
RelΔCO_2_	−1.7% (37.3%)	−0.04% (33.1%)	−10.9% (35.9%)	0.039
PaO_2_	235 (136)	101 (24.2)	189 (128)	<0.001
Lactate	4.36 (4.79)	2.68 (2.68)	3.77 (4.25)	<0.001
PIP	24.0 (6.52)	21.4 (4.56)	23.1 (6.04)	<0.001
SBP	92.5 (20.0)	101 (17.3)	95.5 (19.5)	<0.001
DBP	62.5 (13.9)	66.4 (11.1)	63.9 (13.1)	<0.001
MAP	72.3 (12.4)	77.7 (9.37)	74.2 (11.7)	<0.001
PIP	24.0 (6.52)	21.4 (4.56)	23.1 (6.04)	<0.001
Pre-ECMO-RRT	631 (12.2%)	306 (11.1%)	937 (11.8%)	0.164
Pre-ECMO CA	1885 (36.3%)	980 (35.5%)	2865 (36.0%)	0.466
IABP	1467 (28.3%)	663 (24.0%)	2130 (26.8%)	<0.001
ECMO duration, hours	170 (215)	163 (162)	168 (198)	0.021
SCAI stage				<0.001
B	236 (4.5%)	128 (4.6%)	364 (4.6%)	
C	773 (14.9%)	442 (16.0%)	1215 (15.3%)	
D	2519 (48.6%)	1489 (53.9%)	4008 (50.4%)	
E	1660 (32.0%)	703 (25.5%)	2363 (29.7%)	

Values are mean ± SD, n (%).

BMI, body mass index; AMI, acute myocardial infarction; CHF, congestive heart failure; VT/VF, ventricular tachycardia/ventricular fibrillation, myocarditis; PC, post-cardiotomy; SBP, systolic blood pressure; DBP, diastolic blood pressure; MAP, mean arterial pressure; PIP, peak inspiratory pressure; RelΔCO_2_, relative change in PaCO_2_; RRT, renal replacement therapy; CA, cardiac arrest; IABP, intra-aortic balloon pump; ECMO, extracorporeal membrane oxygenation; SCAI, Society for Cardiovascular Angiography & Interventions.

### Role of the funding source

All the funding of the study had a role in study design, data collection, data analysis, or data interpretation.

## Results

### Patient characteristics

**Fig. 1:**
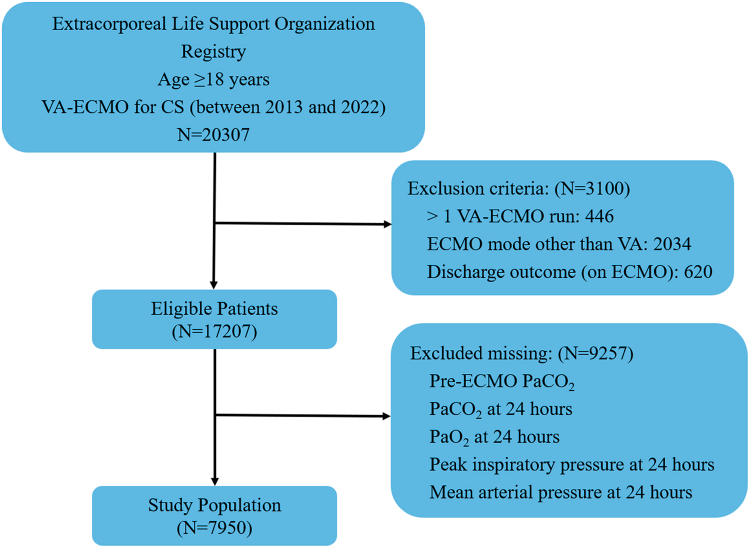
**Study flow.** VA-ECMO, veno-arterial extracorporeal membrane oxygenation; CS, cardiogenic shock.

From an initial pool of 20,307 adult patients who received VA-ECMO for CS between 2013 and 2022, 3100 patients were excluded based on study criteria (446 with >1 VA-ECMO run, 2034 with ECMO mode other than VA, and 620 discharged on ECMO). Additionally, 9257 patients were excluded due to missing critical data (pre-ECMO PaCO_2_, PaCO_2_ at 24 h, PaO_2_ at 24 h, PIP at 24 h, or MAP at 24 h). A total of 7950 adult patients receiving VA-ECMO from 2013 to 2022 were enrolled in the study after exclusions (Fig. 1). The bundle fully achieved in 2762 patients (34.7%), and patient characteristics are described in Table 1. Among patients in the non-bundle group, 0.8% met none of the four bundle components, 18.1% met one component, 61.3% met two components, and 19.8% met three components. Besides, the proportion of patients achieving each individual physiological target was 68.6% for MAP, 22.1% for PaO_2_, 93.4% for RelΔCO_2_, and 16.1% for PIP (Supplementary Table S2). The mean age (SD) in the cohort was 56.5 ± 14.3 years. Male patients accounted for 67.7% of the total population, and their proportion was higher in the bundled group compared with females (69.7% vs. 66.7%; *p* = 0.006). AMI was more common in the bundle group (31.8% vs. 28.7%; *p* = 0.004), whereas PC was more common in the non-bundle group (23.4% vs. 18.8%; *p* < 0.001). Blood gas results were worse in the non-bundle group than in the bundle group, including PH (7.26 ± 0.147 vs. 7.28 ± 0.130), PaCO_2_ (44.8 ± 23.8 vs. 40.9 ± 11.0 mmHg), and lactate levels (8.02 ± 5.61 vs. 6.78 ± 5.03 mmol/L) (all *p* < 0.001). Blood pressures were higher in the bundle group (all *p* < 0.05). ECMO duration was longer in the non-bundle group (170 ± 215 vs. 163 ± 162 h; *p* = 0.021). SCAI stage showed fewer severe CS cases (SCAI stage D/E) in the bundle group (79.4% vs. 80.6%; *p* < 0.001).

### Achievement with bundle

**Fig. 2:**
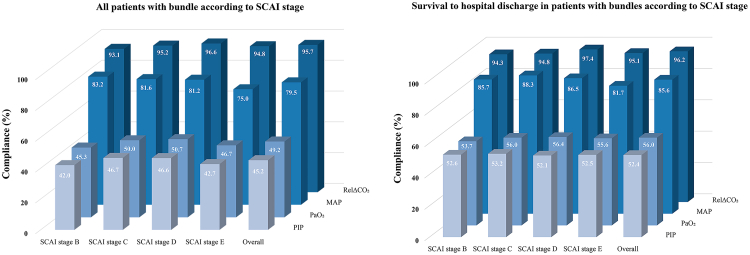
**Achievement of bundle.** MAP, mean arterial pressure; PIP, peak inspiratory pressure; RelΔCO_2_, relative change in PaCO_2_; SCAI, Society for Cardiovascular Angiography & Interventions.

Among the four components of the bundle, overall achievement varied by SCAI stage, ranging from 45.2% to 95.7%. For all patients, the highest achievement was observed for RelΔCO_2_ (93.1%–96.6%) and MAP (75%–83.2%), while the lowest achievement was seen for PIP (42.0%–46.7%) and the oxygenation target (45.3%–50.7%). In patients' survival to hospital discharge, achievement rates were higher across all components, particularly for RelΔCO_2_ (94.3%–97.4%) and MAP (81.7%–88.3%), with improvements also seen for PIP (52.1%–53.2%) and oxygenation target (53.7%–56.4%) (Fig. 2).

### Relative contribution of individual bundle components

**Fig. 3:**
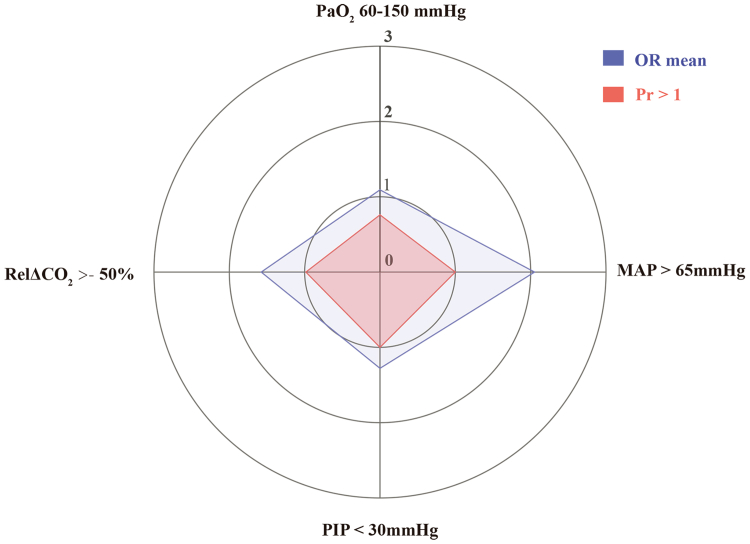
**Radar plot for the four components of bundle.** OR, odds ratio; Pr > 1, probability of outcome > OR of 1 (%); MAP, mean arterial pressure; PIP, peak inspiratory pressure; RelΔCO_2_, relative change in PaCO_2_.

Using Bayesian logistic regression, MAP > 65 mmHg (adjusted odds ratio [aOR] = 2.052, 95% CrI [1.829, 2.311]), RelΔCO_2_ > −50% (aOR = 1.576, 95% CrI [1.271, 1.997]), and PIP < 30 mmHg (aOR = 1.278, 95% CrI [1.018, 1.607]) were the bundle components significantly associated with survival to hospital discharge. Oxygenation target values (PaO_2_) in the range of 60–150 mmHg (aOR = 1.092, 95% CrI [0.856, 1.366]) did not show a statistically significant effect (Supplementary Table S3 and Fig. 2). A radar plot showed the relative contribution of individual bundle components (Fig. 3).

### Optimal bundle of care

**Fig. 4:**
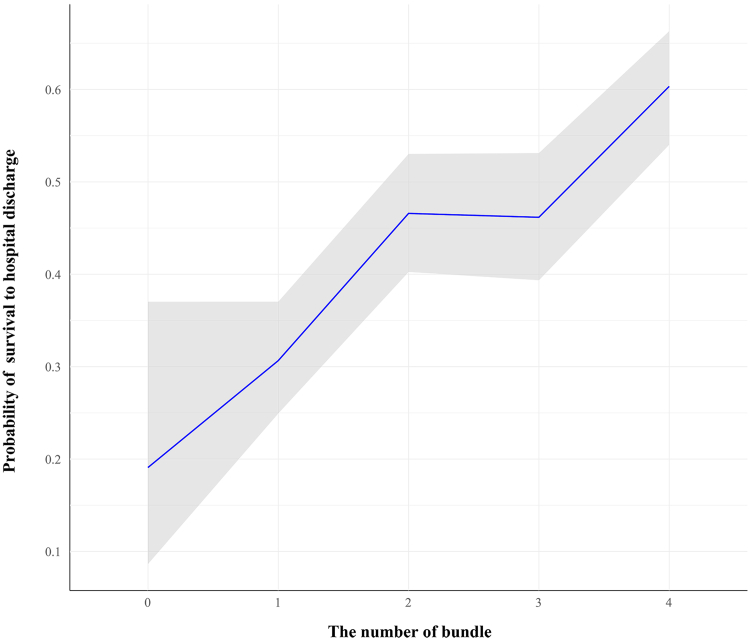
**Relationship between the number of bundle components achieved and probability of survival to hospital discharge.** A Bayesian logistic regression model with a spline function was used to estimate survival probability at each bundle level (0–4). The blue line represents the posterior mean probability of survival; shaded regions represent 95% credible intervals.

**Supplementary Figure S3:**
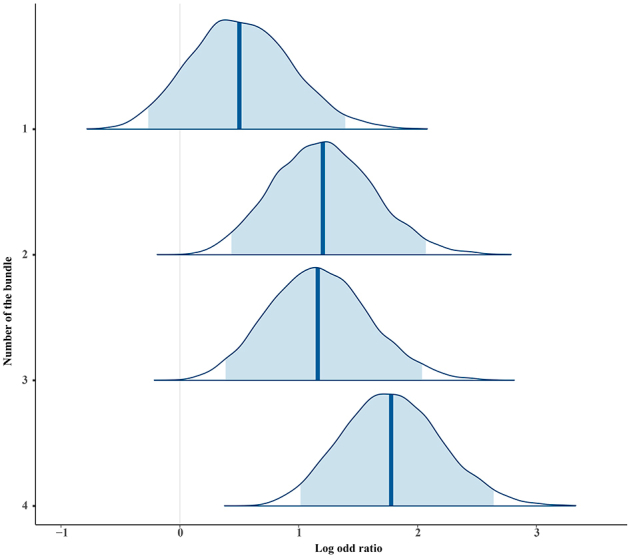


As shown in Fig. 4, the probability of survival increased in a nearly linear fashion with each additional bundle component, particularly between 0 and 2 components, which supports a dose-response relationship between increasing number of bundle parameters achieved and the odds of survival to hospital discharge among patients receiving VA-ECMO. Supplementary Figure S3 depicts the association between the number of bundle components achieved bundle number and survival to hospital discharge. Weak information a priori results showed that the odd ratio increased gradually with the number of parameters, 1 (adjusted log odds ratio [alogOR] 0.52, 95% CrI [−0.30, 1.31]), 2 (alogOR 1.21, 95% CrI [0.41, 2.02]), 3 (alogOR 1.18, 95% CrI [0.35, 1.98]), and 4 (alogOR 1.78, 95% CrI [1.03, 2.60]) (Supplementary Table S4).

### Bundle and primary outcome

**Fig. 5:**
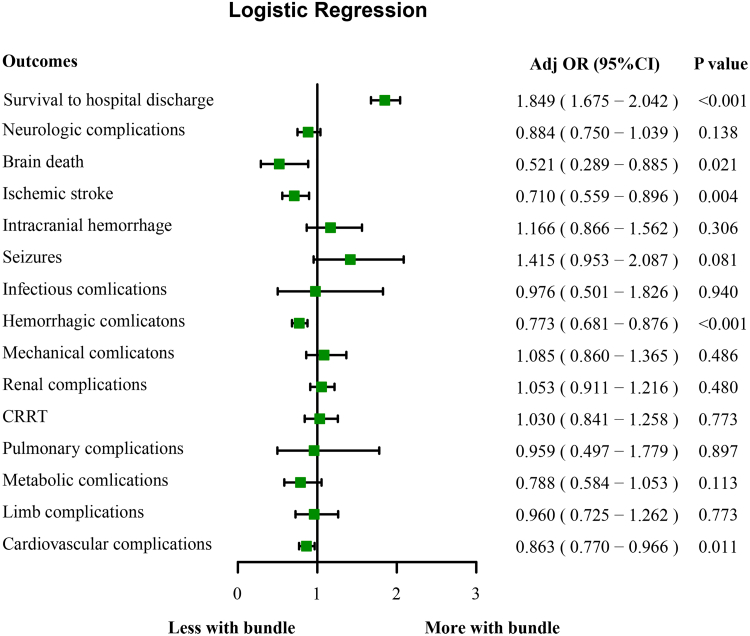
**The relationship between bundle and outcomes.** Adjusted for sex, age, race, weight, pre-ECMO pH, pre-ECMO lactate, pulse pressure at 24 h, diagnoses associated with cardiogenic shock, pre-ECMO support, pre-ECMO cardiac arrest, and SCAI stage. OR, odds ratio; CRRT, continuous renal replacement therapy.

**Fig. 6:**
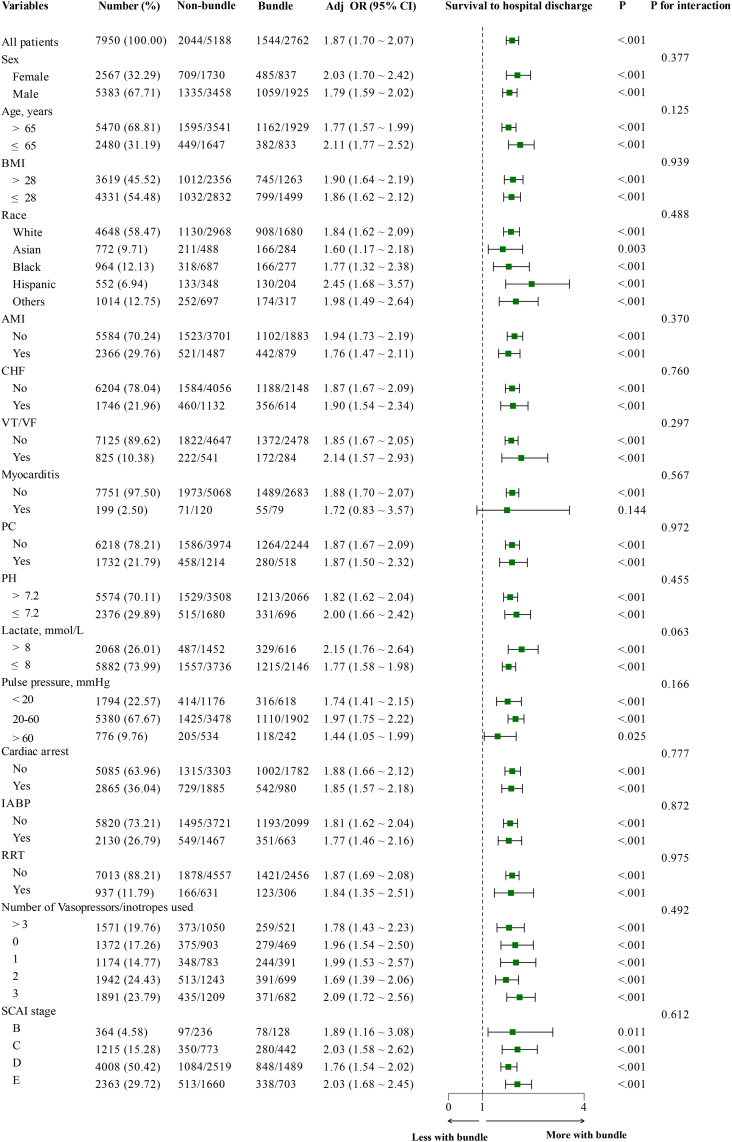
**Subgroup analyses examining the association between bundle and survival to hospital discharge in the unmatched cohort.** Adjusted for sex, age, race, weight, pre-ECMO pH, pre-ECMO lactate, pulse pressure at 24 h, diagnoses associated with cardiogenic shock, pre-ECMO support, pre-ECMO cardiac arrest, and SCAI stage. OR, odds ratio; BMI, body mass index; AMI, acute myocardial infarction; CHF, congestive heart failure; VT/VF, ventricular tachycardia/ventricular fibrillation, myocarditis; PC, post-cardiotomy; RRT, renal replacement therapy; IABP, intra-aortic balloon pump; ECMO, extracorporeal membrane oxygenation; SCAI, Society for Cardiovascular Angiography & Interventions.

**Supplementary Figure S4:**
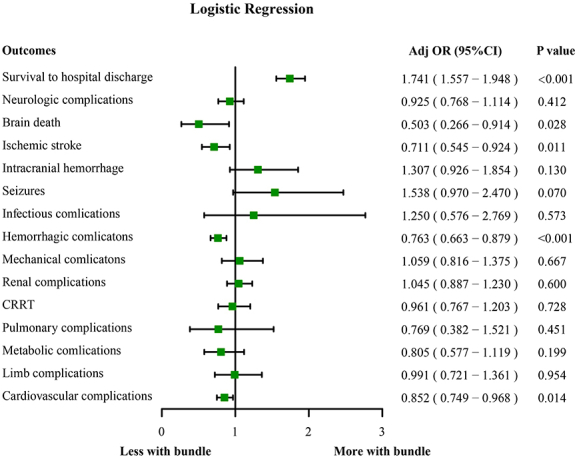


The rate of survival to hospital discharge was higher in the bundle group compared with the non-bundled group (55.9% vs. 39.4%, *p* < 0.001) (Table 2), and multivariable logistic regression modeling demonstrated a statistically significant association between use of the bundle and higher survival to hospital discharge (adjusted odds ratio [aOR] 1.849, 95% CI [1.675, 2.042]; *p* < 0.001) (Fig. 5). After propensity matching in the cohort, the use of the bundle remained statistically significant associated with higher survival to hospital discharge (aOR = 1.741, 95% CI: 1.557–1.948; *p* < 0.001) (Supplementary Figure S4). The association between the bundle and higher discharge alive rates was consistent across all subgroups, including sex (male vs. female), age (younger vs. older), obesity status (with vs. without obesity), race (White, Asian, Black, Hispanic, and others), diagnoses associated with CS (AMI, CHF, VT/VF, myocarditis, and PC), pre-ECMO lactate level (higher vs. lower), pre-ECMO pH level (higher vs. lower), pulse pressure (lower, normal, and higher), pre-ECMO CA (Yes vs. No), IABP (Yes vs. No), RRT (Yes vs. No), types of vasopressors/inotropes (0, 1, 2, 3, and >3), and SCAI stage (B, C, D, and E). No significant interactions were found between the bundle and the variables defining the subgroups (Fig. 6). These findings were consistent with those of the propensity-matched cohort (Supplementary Figure S5).

**Table 2:** Outcomes between the two groups.

Variable	Before propensity matching			After propensity matching		
	Non-bundle (N = 5188)	Bundle (N = 2762)	*p*-value	Non-bundle (N = 2762)	Bundle (N = 2762)	*p*-value
Survival to hospital discharge	2044 (39.4%)	1544 (55.9%)	<0.001	1116 (40.4%)	1544 (55.9%)	<0.001
Complications						
Neurologic	515 (9.9%)	230 (8.3%)	0.023	267 (9.7%)	230 (8.3%)	0.091
Brain death	87 (1.7%)	24 (0.9%)	0.002	44 (1.6%)	24 (0.9%)	0.020
Ischemic stroke	283 (5.5%)	102 (3.7%)	<0.001	140 (5.1%)	102 (3.7%)	0.015
CNS hemorrhage	151 (2.9%)	93 (3.4%)	0.317	76 (2.7%)	93 (3.4%)	0.211
Seizures	64 (1.2%)	46 (1.7%)	0.142	40 (1.4%)	46 (1.7%)	0.587
Infectious	29 (0.6%)	15 (0.5%)	1.000	17 (0.6%)	15 (0.5%)	0.859
Hemorrhagic	1034 (19.9%)	431 (15.6%)	<0.001	536 (19.4%)	431 (15.6%)	<0.001
Mechanical	214 (4.1%)	124 (4.5%)	0.478	121 (4.4%)	124 (4.5%)	0.896
Renal	620 (12.0%)	344 (12.5%)	0.536	331 (12.0%)	344 (12.5%)	0.622
CRRT	304 (5.9%)	163 (5.9%)	0.980	154 (5.6%)	163 (5.9%)	0.644
Pulmonary	29 (0.6%)	15 (0.5%)	1.000	18 (0.7%)	15 (0.5%)	0.727
Metabolic	171 (3.3%)	66 (2.4%)	0.028	82 (3.0%)	66 (2.4%)	0.211
Limb	168 (3.2%)	80 (2.9%)	0.443	78 (2.8%)	80 (2.9%)	0.936
Cardiovascular	1264 (24.4%)	592 (21.4%)	0.004	687 (24.9%)	592 (21.4%)	0.003

CNS, central nervous system; CRRT, continuous renal replacement therapy.

### Bundle and others outcomes

The rate of neurological complications was significantly lower in the group with the bundled intervention than in the unbundled group (8.3% vs. 9.9%; *p* = 0.022), as evidenced by a reduced risk of brain death (0.9% vs. 1.7%; *p* = 0.005) and ischemic stroke (3.7% vs. 5.5%; *p* < 0.001). In addition, the group also presented lower incidence of hemorrhagic (15.6% vs. 19.9%; *p* < 0.001), metabolic (2.4% vs. 3.3%; *p* = 0.028), and cardiovascular complications (21.4% vs. 24.4%; *p* = 0.004), as shown in Table 2. Logistic regression analysis shown that the bundled group was significantly associated with a reduced risk of several complications: a lower risk of brain death (aOR = 0.521, 95% CI: 0.289–0.885; *p* = 0.021) and ischemic stroke (aOR = 0.710, 95% CI: 0.559–0.896; *p* = 0.004). A reduction in the risk of hemorrhagic complications (aOR = 0.773, 95% CI: 0.681–0.876; *p* < 0.001) as well as a reduction in the risk of cardiovascular complications (aOR = 0.863, 95% CI: 0.770–0.966; *p* = 0.011) were also observed (Fig. 5). The propensity-score-matched sensitivity analysis showed robustness of the results (Supplementary Figure S4).

## Discussion

In this large cohort based on a national registry, we found that increasing achievement to this simple bundle within the first 24 h was associated with significantly improved discharge survival. Achievement to bundle varied across the different SCAI stages, with higher achievement to MAP and RelΔCO_2_ and lower achievement to PIP and oxygenation goals. Among patients who survived discharge, increasing achievement to all bundle components was observed. These associations were consistent across patient subgroups and were accompanied by a reduction in many complications.

Several recent studies have highlighted the value of standardized treatment protocols for the prognosis of patients with VA-ECMO, Salazard et al. demonstrated that multidisciplinary treatment packages resulted in a 50% reduction in mortality,^11^ while Jing et al. reported a 35% improvement in survival through standardized hemodynamic management.^12^ Recent publications emphasize the significant heterogeneity of ECMO management in clinical practice and highlight the potential for standardized ECMO management in reducing complications.^13^^,^^14^ In contrast to previous studies evaluating complex intervention protocols, our study employed a simple bundle containing four key evidence-based physiological parameters: MAP > 65 mmHg, PaO_2_ 60–150 mmHg, RelΔCO_2_ > −50%, and PIP < 30 mmHg, which prioritized basic physiological stabilization over complex intervention. The results of this study showed an absolute improvement in survival to hospital discharge of 16.5% in bundle group compared to the non-bundle group, indicating that the treatment package was associated with significantly improved survival.

Previous studies have found that controlled reperfusion neuroprotective management significantly reduces neurological complications, especially ischemic stroke and brain death.^15^ The sepsis treatment bundle has also been shown to reduce hemorrhagic and cardiovascular risks,^16^ indicating that standardized management is generally beneficial for the prognosis of critically ill patients. Although the treatment bundle in this study was effective, there were differences in achievement to the various components of the bundle, suggesting potential challenges to implementation at centers with varying levels of experience and management in VA-ECMO treatment.

Tanaka et al. found that a MAP <70 mmHg is associated with higher mortality, while a MAP >90 mmHg improves survival, highlighting the importance of balancing both low and high perfusion risk.^4^ In this study, the MAP target of >65 mmHg was associated with improved survival, suggesting a protective effect by addressing cerebral autoregulation dysfunction,^17^^,^^18^ This moderate MAP threshold aligns with prior recommendations and may reduce the risk of intracranial hypertension, which has been observed with aggressive MAP targets (>80 mmHg) after the restoration of spontaneous circulation.^19^ By incorporating MAP, oxygenation, and ventilation targets, this study operationalized the hemodynamic-first paradigm advocated by the ELSO guidelines, demonstrating its feasibility in clinical practice.^20^

Studies have highlighted the risks of rapid PaCO_2_ correction, with evidence showing that a >50% reduction in PaCO_2_ within 24 h has been shown to cause cerebral vasoconstriction, reducing cerebral oxygen supply by 32%, and increasing the risk of hypoxic-ischemic brain injury due to pH-driven calcium flux abnormalities.^6^^,^^7^^,^^18^^,^^21^ The bundle in this study limited relative change in PaCO_2_ to >−50%, which resulted in a reduction in neurological complications, particularly brain death and ischemic stroke. Gradual PaCO_2_ normalization likely preserved cerebrovascular reactivity, maintaining cerebral blood flow within autoregulatory thresholds.^22^ Furthermore, the integration of the MAP target (>65 mmHg) ensured adequate perfusion pressure to counteract the vasoconstrictive effects of rapid CO_2_ correction.^18^^,^^23^

Regarding PIP management, elevated PIP exacerbates alveolar strain, which triggers metalloproteinase-9 release and endothelial glycocalyx degradation, leading to capillary leakage and coagulopathy.^24^^,^^25^ By controlling PIP (<30 mmHg), 38% lower mechanical power, reduced ventilator-associated lung injury and systemic inflammation, and helped prevent hyperoxia-related oxidative stress.^26^ Additionally, PIP control improved right ventricular-pulmonary coupling, as evidenced by a reduction in mean pulmonary artery pressure. This benefit was particularly pronounced in patients with pulmonary hypertension and CS.^18^ The reduction in complications observed in this study further supports the multi-system protective effects of standardized PIP management.

Several studies have found that severe hyperoxia (PaO_2_ ≥ 300 mmHg) is strongly associated with increased mortality and neurological complications, which may be due to oxidative stress and reperfusion injury.^5^^,^^27^ Meanwhile, studies have confirmed a dose-dependent relationship between hyperoxia and in-hospital mortality, with severe hyperoxia leading to a 120% increased risk of death.^5^ Shou et al. found that hyperoxia during ECMO resulted in a 59% increase in the incidence of acute brain injury.^27^ In contrast, hypoxia (PaO_2_ < 60 mmHg) may exacerbate systemic ischemia-reperfusion injury.^28^ The oxygenation target (PaO_2_ between 60 and 150 mmHg) in this bundle directly addresses these risks, providing a physiological balance consistent with the ELSO guidelines.^29^ The significant reduction in neurological complications in the bundle group reflects optimized cerebral oxygenation. Mechanistic studies indicate that hyperoxia disrupts cerebral autoregulation, increases free radical production, and promotes blood-brain barrier permeability. Standardized oxygenation management within the first 24 h in this study, during the critical window for mitigating reperfusion injury, improved prognosis and confirmed the value of integrating oxygenation targets into early care bundle.^28^

Despite the benefits of the bundle, full achievement was achieved by only 34.7% of patients, reflecting challenges similar to those with lung-protective ventilation protocols, where <40% met the targets.^24^ Potential barriers to achievement may include insufficient real-time PaCO_2_ monitoring (absent in 62% of non-adherent cases), leading to a higher rapid CO_2_ correction rate.^21^ Furthermore, variability in oxygenation management may stem from conflicting institutional practices and challenges in consistent monitoring approaches during VA-ECMO support.^30^ However, studies have shown that bundle-adherent patients demonstrated significantly better outcomes, with 98% achieving ≥3 target achievements, compared to 29% in non-adherent patients. Achievement was associated with improved survival. Non-adherent patients were more likely to exceed safe PaCO_2_ correction limits and tolerate harmful PIP levels, increasing the risk of complications.^6^^,^^24^ These results emphasize the need for better monitoring and ECMO team coordination to improve achievement and outcomes in VA-ECMO patients.

Our study has a number of important limitations. Firstly, as a retrospective cohort study, selection bias was inevitable, particularly in patient grouping and treatment selection. We recognize that the ability to achieve bundle components may reflect overall patient severity or favorable early trajectory rather than the causal effect of the bundle itself. Although our analyses adjusted for multiple indicators of illness severity and used propensity score matching, residual confounding is possible. In addition, plateau pressure is a more accurate indicator of lung protective ventilation, but this parameter is not available in the ELSO registry. Therefore, the use of PIP as a metric has limitations in reflecting true alveolar pressure. Furthermore, while multiple imputation addressed missing data, incomplete clinical recording could still introduce bias. Additionally, the lack of long-term follow-up limits our ability to evaluate the sustained effects of the bundle on survival and complications. Moreover, more than half of the initially eligible population was excluded from this study due to missing data on key bundle components. Although the final sample size remained substantial, this exclusion may have introduced selection bias and limited the generalizability of the findings. Therefore, future prospective, randomized controlled trials with long-term follow-up are needed to validate these findings and optimize VA-ECMO management.

In summary this large registry-based cohort, shows a simple bundle within the first 24 h—targeting MAP >65 mmHg, limiting PaCO_2_ reduction (RelΔCO_2_ > −50%), using lung-protective ventilation (PIP < 30 mmHg), and maintaining balanced oxygenation (PaO_2_ 60–150 mmHg)—has the potential to significantly improved survival to hospital discharge and reduced complications in VA-ECMO patients with CS. Future research should focus on randomized controlled trials to evaluate the real world relevance and long-term outcomes of VA-ECMO in CS, which would provide robust evidence for personalized treatment strategies and enhance clinical decision-making.

## Contributors

Liangshan Wang and Xiaotong Hou conceptualized the study. Feng Yang, Chenglong Li, Xing Hao, Zhongtao Du, and Hong Wang participated in methodology. Liangshan Wang, Chenglong Li, Xing Hao, Zhongtao Du, and Xiaotong Hou contributions were funding acquisition. Liangshan Wang, Kexin Wang, and Yan Wang performed formal analysis. Liangshan Wang and Kexin Wang were contributors in writing the original draft. All authors were contributors in reviewing and revising the manuscript. Liangshan Wang and Xiaotong Hou had access to and verified the underlying data. All authors read and approved the final version of the manuscript.

## Data sharing statement

The data that support the findings of this study are available from the corresponding author upon reasonable request. Access to the data is subject to approval by the ELSO registry.

## Declaration of interests

All authors declare no competing interests related to this study.

## Figures and Tables

**Supplementary Figure S2:**
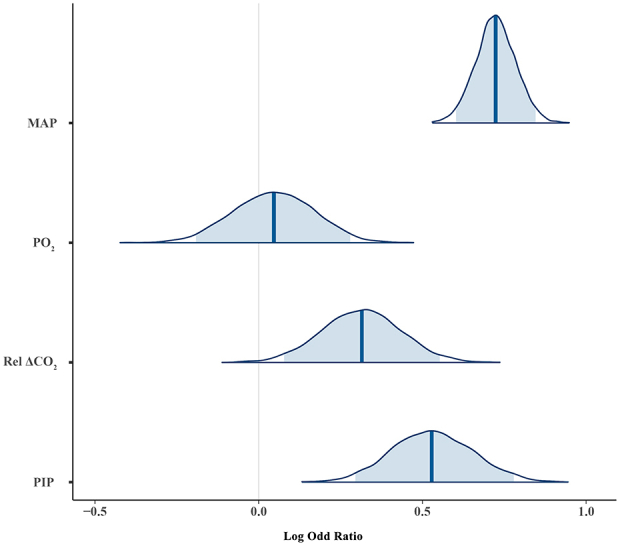


**Supplementary Figure S5:**