# Supplementary material for: A simple VA-ECMO bundle in adult patients with cardiogenic shock: an analysis of ELSO registry
Source: eClinicalMedicine. 2025 Aug 7;87:103423. doi: 10.1016/j.eclinm.2025.103423 (PMC12355413; doi:10.1016/j.eclinm.2025.103423)
Supplement: Supplementary Materials [file mmc1.docx]

**Supplementary material**

**Supplementary Methods**

**Bayesian Logistic Regression Model Details**

For the Bayesian logistic regression model used to identify optimal bundle components, we employed a carefully designed analytical approach. The prior distribution for the regression coefficients was specified as a Student-t distribution with 3 degrees of freedom, a mean of 0, and a scale of 2.5. This represents a weakly informative prior, commonly recommended in Bayesian logistic regression, which avoids excessive shrinkage while stabilizing estimates—particularly in the context of potentially weak associations. A scale of 2.5 corresponds to a wide, plausible range of odds ratios (approximately 1/12 to 12), allowing the data to drive posterior inference. Posterior inference was conducted using the Markov Chain Monte Carlo (MCMC) method to estimate the posterior distributions of the model parameters. The MCMC sampling involved 4 chains, each running 2000 iterations, with the first 1000 iterations used as warmup. The effect of each parameter on survival to hospital discharge was assessed by calculating its posterior mean and 95% credible interval (CrI). This probability (denoted as Pr > 1) provides an interpretable Bayesian measure of the likelihood that a given bundle component is associated with improved outcome.. Additionally, a posterior prediction check was performed to verify model robustness and assess MCMC chain convergence, demonstrating that the model performed well and the fit accuracy was confirmed by the prediction check. The bundle included the following variables and corresponding value ranges, measured at 24 hours of ECMO: MAP > 65 mmHg, PaO_2_ 60-150 mmHg, PIP < 30 mmHg, and Rel∆CO_2_ >-50%). The selection model was adjusted by gender, age, race, weight, diagnoses associated with CS (including acute myocardial infarction [AMI], congestive heart failure [CHF], ventricular tachycardia/ventricular fibrillation [VT/VF], myocarditis, and post-cardiotomy [PC]), pre-ECMO support, and cardiac arrest (CA). To examine the dose–response relationship between the number of bundle components achieved and the probability of survival to hospital discharge, we constructed the Bayesian logistic regression model incorporating a tensor product spline function for the bundle count variable (ranging from 0 to 4). This spline-based approach allowed for flexible modeling of non-linear effects. The model showed that achievement of all four components was associated with significantly improved survival to hospital discharge compared to partial or non-achievement of the bundle components. The optimal bundle combination was determined by evaluating different combinations of these components and selecting the set that demonstrated the strongest association with survival to hospital discharge.

**Detailed Complication Categories and Definitions**

The secondary outcomes included the following complication categories, including neurologic (any of the following: brain death, central nervous system [CNS] diffuse ischemia by CT/MRI, CNS hemorrhage by US/CT, CNS infarction by US/CT/MRI, intra/extra parenchymal and intraventricular CNS hemorrhage by US/CT/MRI, neurosurgical intervention performed, and seizures confirmed by EEG/clinically determined), infectious (any of the following: culture proven infection and white blood cell count < 1,500), hemorrhagic (any of the following: peripheral/mediastinal cannulation site bleeding, cannulation and surgical site bleeding, gastrointestinal hemorrhage, hemolysis: hemoglobin > 50 mg/dl, and disseminated intravascular coagulation), mechanical (any of the following: air in circuit, cannula problems, circuit change, clots and air emboli, clots: hemofilter, cracks in pigtail connectors, other tubing rupture, oxygenator failure, pump failure, raceway rupture, and thrombosis/clots: circuit component), renal (any of the following: creatinine levels between 1.0-3.0 mg/dL, creatinine > 3.0 mg/dL, and the need for renal replacement therapy), metabolic (any of the following: severe hemolysis, moderate hemolysis, hyperbilirubinemia, glucose < 40 mg/dL, glucose > 240 mg/dL, PH < 7.2, and PH > 7.6), limb (any of the following: ischemia, fasciotomy, compartment syndrome, and amputation) and cardiovascular (any of the following: cardiac arrhythmia, tamponade [blood], patent ductus arteriosus: left to right, myocardial stun, hypertension requiring vasodilators, inotropes on ECLS, CPR required) complications.

**Supplementary Tables**

**Supplementary Table 1 Demographic and clinical characteristics of the PSM cohort**

| **Variable Mean (SD) / n (%)** | **Non-bundle (n=2762)** | **Bundle (n=2762)** | **Std. Mean Diff.** | **p-value** |
| --- | --- | --- | --- | --- |
| **Age, years** | 56.55 (14.49) | 56.57 (13.83) | 0.002 | 0.955 |
| **Gender (male)** | 1933 (70.0) | 1925 (69.7) | 0.006 | 0.837 |
| **Race (%)** |  |  | 0.026 | 0.918 |
| White | 1681 (60.9) | 1680 (60.8) | - | - |
| Asian | 273 (9.9) | 284 (10.3) | - | - |
| Black | 265 (9.6) | 277 (10.0) | - | - |
| Hispanic | 211 (7.6) | 204 (7.4) | - | - |
| Others | 332 (12.0) | 317 (11.5) | - | - |
| **Year (%)** |  |  | 0.024 | 0.663 |
| 2013-2016 | 152 (5.5) | 142 (5.1) | - | - |
| 2017-2019 | 1148 (41.6) | 1128 (40.8) | - | - |
| 2020-2022 | 1462 (52.9) | 1492 (54.0) | - | - |
| **Weight, kg** | 86.95 (23.17) | 87.27 (22.00) | 0.014 | 0.592 |
| **Height, cm** | 171.70 (11.60) | 171.84 (11.35) | 0.013 | 0.639 |
| **AMI** | 896 (32.4) | 879 (31.8) | 0.013 | 0.645 |
| **CHF** | 622 (22.5) | 614 (22.2) | 0.007 | 0.821 |
| **PC** | 491 (17.8) | 518 (18.8) | 0.025 | 0.365 |
| **VT/VF** | 295 (10.7) | 284 (10.3) | 0.013 | 0.660 |
| **Myocarditis** | 77 (2.8) | 79 (2.9) | 0.004 | 0.935 |
| **Other diagnoses** | 381 (13.8) | 388 (14.0) | 0.007 | 0.816 |
| **Pre-ECMO CA** | 969 (35.1) | 980 (35.5) | 0.008 | 0.778 |
| **Pre-ECMO RRT** | 297 (10.8) | 306 (11.1) | 0.010 | 0.730 |
| **IABP** | 676 (24.5) | 663 (24.0) | 0.011 | 0.706 |
| **Type of vasopressors** |  |  | 0.024 | 0.941 |
| 0 | 460 (16.7) | 469 (17.0) | - | - |
| 1 | 375 (13.6) | 391 (14.2) | - | - |
| 2 | 695 (25.2) | 699 (25.3) | - | - |
| 3 | 700 (25.3) | 682 (24.7) | - | - |
| >3 | 532 (19.3) | 521 (18.9) | - | - |
| **ECMO duration, hours** | 161.62 (150.00) | 163.03 (161.75) | 0.009 | 0.737 |
| **Pre-ECMO PH** | 7.28 (0.14) | 7.28 (0.13) | 0.004 | 0.888 |
| **pre-ECMO lactate，mmol/L** | 6.24 (4.66) | 6.10 (4.66) | 0.029 | 0.285 |
| **SCAI stage** |  |  | 0.038 | 0.565 |
| B | 120 (4.3) | 128 (4.6) | - | - |
| C | 409 (14.8) | 442 (16.0) | - | - |
| D | 1506 (54.5) | 1489 (53.9) | - | - |
| E | 727 (26.3) | 703 (25.5) | - | - |

PSM, propensity score matching; AMI, acute myocardial infarction; CHF, congestive heart failure; VT/VF, ventricular tachycardia/ventricular fibrillation, myocarditis; PC, post-cardiotomy; CA, cardiac arrest; RRT, renal replacement therapy; IABP, intra-aortic balloon pump; ECMO, extracorporeal membrane oxygenation; SCAI, Society for Cardiovascular Angiography & Interventions

**Supplementary Table 2** Achievement with bundle component in the non-bundle Group

|  | **Non-bundle (N=5188)** |
| --- | --- |
| **Number of bundle component achieved** |  |
| 0 | 39 (0.8%) |
| 1 | 941 (18.1%) |
| 2 | 3179 (61.3%) |
| 3 | 1029 (19.8%) |
| **Bundle component** |  |
| MAP > 65 mmHg | 3559 (68.6%) |
| PaO_2_ 60 - 150 mmHg | 1147 (22.1%) |
| RelΔCO₂ > -50% | 4845 (93.4%) |
| PIP < 30 cmH₂O | 835 (16.1%) |

MAP, mean arterial pressure; Rel∆CO_2_, relative change in PaCO_2_; PIP, peak inspiratory pressure.

**Supplementary Table 3 Relative contribution of individual bundle components**

| **Variable** | **Adj OR** | **95% CrI** | **Pr > 1** |
| --- | --- | --- | --- |
| MAP > 65mmHg | 2.052 | 1.829-2.311 | > 99 % |
| PaO_2_ 60-150mmHg | 1.092 | 0.856-1.366 | 76.0% |
| Rel∆CO_2_ > -50% | 1.576 | 1.271-1.997 | 98.4 % |
| PIP < 30mmHg | 1.278 | 1.018-1.607 | >99 % |

OR odds ratio, CrI credible interval, Pr > 1 probability of outcome > OR of 1 (%); MAP, mean arterial pressure; Rel∆CO_2,_ relative change in PaCO_2_; PIP, peak inspiratory pressure. Adjusted OR by gender, age, race, weight, diagnoses associated with cardiogenic shock (including acute myocardial infarction, congestive heart failure, ventricular tachycardia/ventricular fibrillation, myocarditis, and post-cardiotomy), pre-ECMO support, and pre-ECMO cardiac arrest.

**Supplementary Table 4 Model results showing association between the number of bundles with survival to hospital discharge**

| **Variable** | **LogOR** | **95% CrI** | **Pr >1** |
| --- | --- | --- | --- |
| 1 component | 0.52 | -0.30 ~ 1.31 | 89% |
| 2 components | 1.21 | 0.41 ~ 2.02 | >99% |
| 3 components | 1.18 | 0.35 ~ 1.98 | >99% |
| 4 components | 1.78 | 0.94 ~ 2.60 | >99% |

OR odds ratio, CrI credible interval, Pr >1 probability of outcome > OR of 1 (%)

The model was adjusted by gender, age, race, weight, diagnoses associated with cardiogenic shock (including acute myocardial infarction, congestive heart failure, ventricular tachycardia/ventricular fibrillation, myocarditis, and post-cardiotomy), pre-ECMO support, and pre-ECMO cardiac arrest.

**Supplementary Table 5 Comparison between multiple imputation and complete case analysis**

| **Variable** | **Multiple Imputation (OR [95% CI]; *P*)** | **Complete Case Analysis (OR [95% CI]; *P*)** |
| --- | --- | --- |
| Bundle | 1.92 [1.73–2.12]; *P* < 0.001 | 1.79 [1.56–2.06]; *P* < 0.001 |
| Age | 0.97 [0.96–0.97]; *P* < 0.001 | 0.97 [0.96–0.97]; *P* < 0.001 |
| Male | 1.06 [0.95–1.18]; *P* = 0.29 | 0.97 [0.83–1.10]; *P* = 0.70 |
| Asian | 1.18 [0.99–1.40]; *P* = 0.06 | 1.21 [0.94–1.51]; *P* = 0.13 |
| Black | 1.15 [0.99–1.34]; *P* = 0.07 | 1.19 [0.94–1.50]; *P* = 0.15 |
| Hispanic | 0.96 [0.80–1.17]; *P* = 0.70 | 1.00 [0.77–1.29]; *P* = 0.99 |
| Others | 0.89 [0.77–1.03]; *P* = 0.12 | 0.97 [0.78–1.19]; *P* = 0.77 |
| Weight | 0.99 [0.99–1.00]; *P* < 0.001 | 0.99 [0.99–1.00]; *P* = 0.13 |
| Acute myocardial infarction | <0.001; wide CI; *P* > 0.95 | <0.001; wide CI; *P* > 0.95 |
| Congestive heart failure | <0.001; wide CI; *P* > 0.95 | <0.001; wide CI; *P* > 0.95 |
| Ventricular tachycardia/fibrillation | <0.001; wide CI; *P* > 0.95 | <0.001; wide CI; *P* > 0.95 |
| Myocarditis | <0.001; wide CI; *P* > 0.95 | <0.001; wide CI; *P* > 0.95 |
| Post-cardiotomy | <0.001; wide CI; *P* > 0.95 | <0.001; wide CI; *P* > 0.95 |
| Others | <0.001; wide CI; *P* > 0.95 | <0.001; wide CI; *P* > 0.95 |
| Pre-ECMO cardiac arrest | 1.01 [0.91–1.12]; *P* = 0.84 | 1.11 [0.94–1.31]; *P* = 0.18 |
| Pre-ECMO RRT | 0.50 [0.42–0.58]; *P* < 0.001 | 0.45 [0.37–0.55]; *P* < 0.001 |
| Pre-ECMO lactate | 0.95 [0.94–0.97]; *P* < 0.001 | 0.95 [0.93–0.97]; *P* < 0.001 |
| PH | 1.77 [1.22–2.58]; *P* = 0.003 | 1.74 [0.96–2.43]; *P* = 0.068 |
| Pulse pressure at 24 hours | 1.01 [1.01–1.01]; *P* < 0.001 | 1.01 [1.01–1.01]; *P* < 0.001 |
| SCAI stage | 1.03 [0.94–1.13]; *P* = 0.49 | 0.89 [0.77–1.03]; *P* = 0.15 |

ECMO, extracorporeal membrane oxygenation; RRT, renal replacement therapy; SCAI, Society for Cardiovascular Angiography & Interventions

**Supplementary Figure legends**

**Supplementary Figure 1 Standardized mean differences before and after propensity score matching**

**Supplementary Figure 2 Posterior distribution of the effects of bundle components on survival to hospital discharge**

MAP, mean arterial pressure; Rel∆CO_2_, relative change in PaCO_2_; PIP, peak inspiratory pressure

**Supplementary Figure 3 Posterior distribution of the effect of the number of bundled components on survival to hospital discharge**

Adjusted for gender, age, race, weight, diagnoses associated with cardiogenic shock, pre-ECMO support, pre-ECMO cardiac arrest.

**Supplementary Figure 4 The relationship between bundle and outcomes in the PSM cohort**

PSM, propensity score matching. Adjusted for gender, age, race, weight, pre-ECMO pH, pre-ECMO lactate, pulse pressure at 24 hours, diagnoses associated with cardiogenic shock, pre-ECMO support, pre-ECMO cardiac arrest, and SCAI stage.

**Supplementary Figure 5 Subgroup analyses examining the association between bundle and survival to hospital discharge in the PSM cohort**

PSM, propensity score matching; OR odds ratio, BMI, body mass index; AMI, acute myocardial infarction; CHF, congestive heart failure; VT/VF, ventricular tachycardia/ventricular fibrillation, myocarditis; PC, post-cardiotomy; RRT, renal replacement therapy; IABP, intra-aortic balloon pump; ECMO, extracorporeal membrane oxygenation; SCAI, Society for Cardiovascular Angiography & Interventions. Adjusted for gender, age, race, weight, pre-ECMO pH, pre-ECMO lactate, pulse pressure at 24 hours, diagnoses associated with cardiogenic shock, pre-ECMO support, pre-ECMO cardiac arrest, and SCAI stage.
